# Reduction of maternal mortality due to preeclampsia in Colombia-an interrupted time-series analysis

**Published:** 2014-03-30

**Authors:** Julián A Herrera, Rodolfo Herrera-Medina, Juan Pablo Herrera-Escobar, Aníbal Nieto-Díaz

**Affiliations:** 1. Director WHO Collaborative Centre for Research in Human Reproduction, Universidad del Valle, Cali, Colombia.; 2. Clinical Professor, Department of Obstetrics & Gynecology, Universidad de Alcalá, Madrid, Spain.; 3. Researcher, WHO Collaborative Centre for Research in Human Reproduction, Universidad del Valle, Cali, Colombia.; 4. Head Professor, Department of Obstetrics and Gynecology, Faculty of Medicine, Universidad de Murcia, Spain.

**Keywords:** Preeclampsia, maternal mortality, population study, bio-psychosocial model

## Abstract

**Introduction:**

Preeclampsia is the most important cause of maternal mortality in developing countries. A comprehensive prenatal care program including bio-psychosocial components was developed and introduced at a national level in Colombia. We report on the trends in maternal mortality rates and their related causes before and after implementation of this program.

**Methods:**

General and specific maternal mortality rates were monitored for nine years (1998-2006). An interrupted time-series analysis was performed with monthly data on cases of maternal mortality that compared trends and changes in national mortality rates and the impact of these changes attributable to the introduction of a bio-psychosocial model. Multivariate analyses were performed to evaluate correlations between the interventions.

**Results:**

Five years after (2002 - 2006) its introduction the general maternal mortality rate was significantly reduced to 23% (OR=0.77, CI 95% 0.71-0.82).The implementation of BPSM also reduced the incidence of preeclampsia in 22% (OR= 0.78, CI 95% 0.67-0.88), as also the labor complications by hemorrhage in 25% (OR=0.75, CI 95% 0.59-0.90) associated with the implementation of red code. The other causes of maternal mortality did not reveal significant changes. Biomedical, nutritional, psychosocial assessments, and other individual interventions in prenatal care were not correlated to maternal mortality (*p*= 0.112); however, together as a model we observed a significant association (*p*= 0.042).

**Conclusions:**

General maternal mortality was reduced after the implementation of a comprehensive national prenatal care program. Is important the evaluation of this program in others populations.

## Abstract (es)

**Introducción:**

La preeclampsia es la causa más importante de mortalidad materna en los países en desarrollo. Se desarrolló e introdujo en Colombia y a nivel nacional un programa de cuidado prenatal que incluyó componentes bio-psicosociales. Se muestran las tendencias de las tasas de mortalidad materna y las causas relacionadas antes y después de la implementación del programa.

**Métodos:**

Las tasas de mortalidad general y específica fueron monitoreadas durante 9 años (1998-2006). Un análisis de series de tiempo ininterrumpido se realizó con datos mensuales de casos de mortalidad materna y se compararon las tendencias de cambio en las tasas de mortalidad nacional y el impacto de los cambios atribuibles a la introducción del modelo. Se hizo un análisis multivariado para evaluar la asociación entre las intervenciones.

**Resultados:**

Cinco años después de la introducción del programa (2002-2006) la tasa de mortalidad maternal se redujo significativamente en 23% (OR=0.77, CI 95% 0.71-0.82). La implementación del MBPS redujo la incidencia de la preeclampsia en 22% (OR= 0.78, CI 95% 0.67-0.88), igual la mortalidad materna por complicación del parto por hemorragias en 25% (OR=0.75, CI 95% 0.59-0.90). Otras causas de mortalidad materna no mostraron cambios significativos. No se correlacionó la mortalidad materna con intervenciones de cuidado prenatal individuales como las biomédicas, nutricionales y psicosociales (*p*= 0.112), sin embargo, ellas en conjunto como un modelo (biopsicosocial) si observaron una asociación significativa (*p*= 0.042).

**Conclusiones:**

La mortalidad maternal general se redujo después de la implementación de un programa nacional de cuidado prenatal. Es importante evaluar este programa en otras poblaciones.

## Introduccion

In developing countries, maternal mortality is a national public health problem 1, and reducing it is a millennium development goal. The social and family life implications, the hospital financial costs and the therapeutic management repercussions are very important points to consider in developing countries.

To achieve this goal, a comprehensive prenatal care program based on the bio-psychosocial model (BPSM) was developed, introduced, and implemented as a national standard of healthcare in Colombia. The BPSM was previously reported to impact obstetric and psychosocial risk factors reducing maternal morbidity and mortality 2 ^,^ 3. 

The first population trial was performed in the western region of Colombia (1995-1997) (n= 14,354) 4. The implementation of the BPSM in this region was associated with lower maternal mortality rates (range: 60-63 x 10^5 ^live newborns) as compared with the remaining geographic regions of the country during the same time (range: 74-100). Similar results were reproduced and validated in national and international trials 5 ^-^ 8.

The baseline data for maternal mortality in the country was gathered during 1998-1999. In 2000, the government promulgated the standard obstetric protocols for the reduction of maternal mortality at the national level (Resolution 412, April 2000, Ministry of Health and Social Protection). In 2001-2002, health teams throughout the country were trained on the BPSM. During 2002-2006 the BPSM model was introduced as public health policy at the national level.

To date, the effectiveness of this national program on maternal mortality is not known. This paper reports the trends and changes in maternal mortality rates before and after introduction and implementation of the BPSM at the national level.

## Material and Methods

A quasi experimental study was performed. The Colombian Ministry of Health and Social Protection launched the norms, regulations, adopted the model, and trained the health teams. Adequate implementation of BPSM was verified through official surveillance which also reported coverage on the application of the protocols.

Maternal mortality had a dual notification system for epidemiological surveillance of maternal deaths (SIVIGILA system and vital statistics). Since 1992, the country has had an official quality assurance program to ensure good medical care with surveillance of this program provided by the Ministry of Social Protection. The most frequent causes of maternal mortality were evaluated: preeclampsia, eclampsia, HELLP syndrome, postpartum hemorrhage, abruption of placenta, placenta preview, sepsis, complications during labor, and embolism.

Actions to improve training among health professionals in rural locations, a decrease in the number of pregnancies lacking medical attention, improved institutional attention during deliveries, improved access to antenatal care, and implementation of a red code for postpartum hemorrhage were all implemented since 2000 in accordance with standard obstetric protocols (Resolution 412, April 2000, Ministry of Health and Social Protection).

The programmatic strategy to reduce preeclampsia risk included BPSM for ambulatory care, and best medical practices during hospital care in accordance with standard obstetric protocols. The introduction of BPSM was directly supervised by the Ministry of Health and Social Protection. The application of BPSM included obstetric, nutritional, and psychosocial interventions as elements in an innovative and integrative model 2 ^-^ 8.

The first introductory step was to train health teams to more fully understand the government guidelines and the methodology of the model (2000-2001). During program introduction, a book concerning the methodology (First Edition, 7300 books ISBN 958-670-095-X) was provided along with scientific support, paper copies of the format for gathering information, and BIOPSICO software to alert professionals regarding obstetric and bio-psychosocial risks. The software was also used to record all information regarding interventions along with the maternal and perinatal outcomes. 

In at least one follow-up meeting, a second edition of the book (3100, ISBN 958-33-3447-2) was provided, he methodology was reinforced, questions were addressed, and the implementation of the software was evaluated. The books were introduced by the Ministry of Social Protection which included a specific declaration of ethical approbation. Informed consent was not required as BPSM was a national public health policy. A third edition of the book was later provided for the implementation of BPSM (1000 books ISBN 978-958-670-716-9) and provided all prophylactic and medical treatments for specific intervention for pregnant women identified with high risk to develop preeclampsia (2002-2006) (Table 1).

**Table 1.**
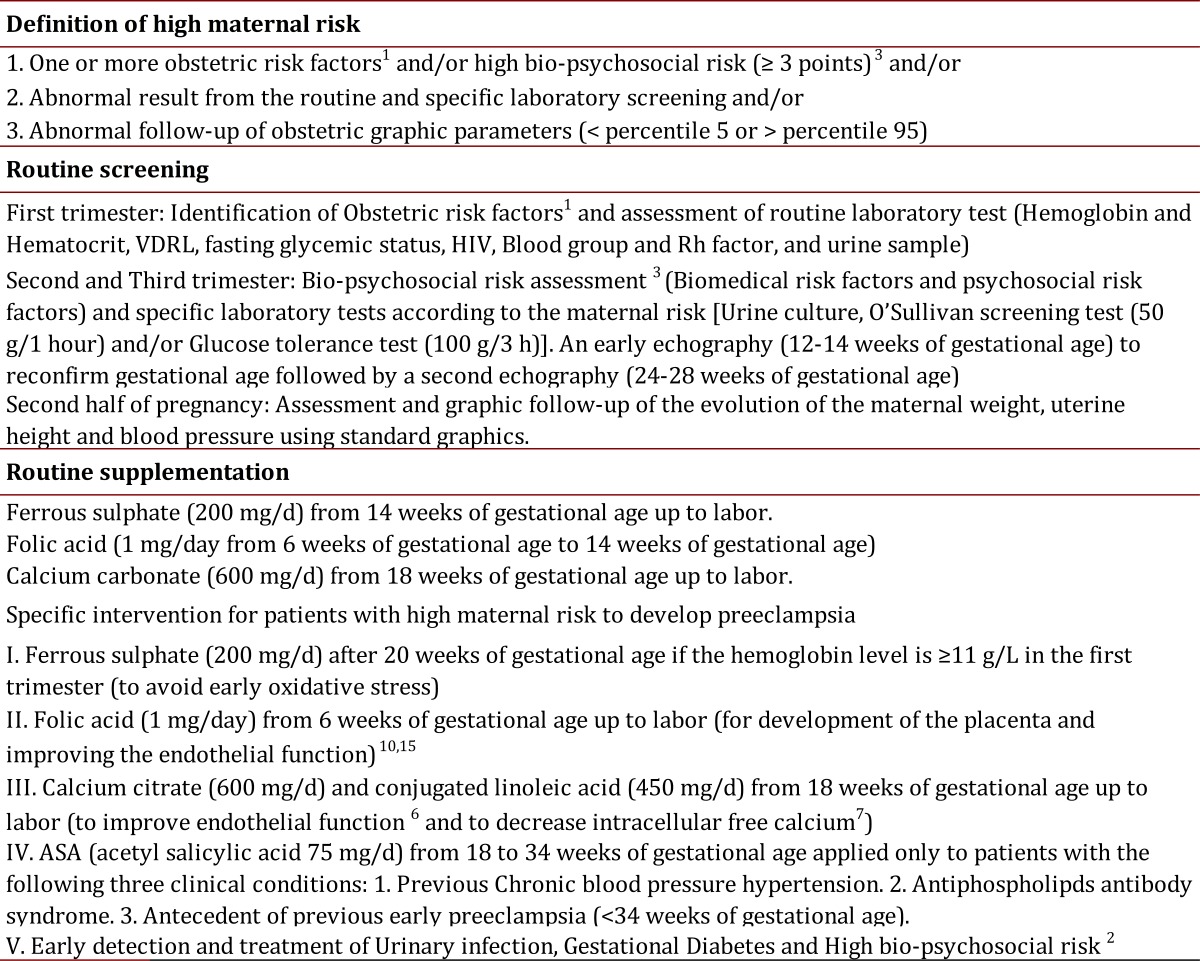
Description of a biopsychosocial model for the intervention of prenatal risk. Colombia 2002-2006

Gestational hypertension was defined as pregnancy-induced hypertension (≥ 140/90 mm Hg) after twenty weeks of gestation without proteinuria (< 300 mg/24 h)^1^. Preeclampsia was defined as pregnancy-induced hypertension with significant proteinuria (≥ 300 mg/ 24 h) 1. Eclampsia was defined as preeclampsia with seizures 1. HELLP syndrome was defined as pregnancy-induced hypertension with hemolysis, elevated liver enzymes, and a low platelet count 1. Maternal mortality (MM) was defined as maternal death during pregnancy, birth and forty days in puerperium, and not associated with incidental or accidental causes.

### Data collection:

The National Department of Statistics (DANE) (www.dane.gov.co/vitalstatistics) collected data on maternal mortality. An epidemiological surveillance committee (COVE) analyzed all maternal deaths. This committee was legislatively established and was composed of the physician in charge of the patient, independent academic gynecologists, epidemiologists, and government officials. This committee analyzed each clinical record, autopsy record, and verbal autopsy to determine the cause of maternal death and associated factors. 

### Statistical methods:

An interrupted time-series analysis was designed and conducted to analyze the Colombian national maternal mortality data between 1998 and 2006. Using monthly data for those years in cases of maternal mortality, we were able to compare trends and changes in national maternal mortality rates. Specifically, we used the techniques developed by Box and Jenkins 9, often referred to as the ARIMA models. In this case, an ARIMA impact analysis was used. Intervention components (dummy variables) were constructed to test whether there was any impact of the intervention that could not be accounted for by normal fluctuations and long-term trends. If the C mistake is high there are any evidence that the intervention affected the time-series ((1)/σ^2^; σ^2^ = residual variance before of the intervention and after intervention).

The maternal mortality rate is defined as the ratio of the number of maternal deaths per 100,000 live births. The general maternal mortality rates included all causes and specific maternal mortality rates were calculated for the most common causes of death (i.e. preeclampsia, HELLP syndrome, complications of labor, hemorrhage, embolism, sepsis and thrombophilia). 

To determine the appropriate application of the BPSM, we evaluated the proportion of pregnant patients at high risk, those receiving calcium supplements, pregnant women receiving treatment for urinary tract infection and/or gestational diabetes, and those receiving social support when high levels of anxiety were identified.

For the multivariate analysis, maternal mortality was the dependent variable. The independent variables were antenatal care during the first trimester, obstetric, nutritional and psychosocial risk interventions. Multiple linear regressions were performed to test the correlations between early prenatal care and the interventions for malnutrition, psychosocial risk and gestational diabetes. Correlation coefficients at their 95% confidence intervals were calculated by using the statistical package SPSS^®^ for Windows (version 20). A *p*= <0.05 was regarded as statistically significant.

## Results

All pregnant women in antenatal care were included in the study. Table 2 shows socio- demographic and obstetric characteristics for every category. Seventy-four percent of the pregnant women received all protocols designed to prevent the most common causes of maternal morbidity and mortality. The adequate introduction of BPSM was confirmed with the rate of calcium supplementation (range 56.8%-97.1%) for the study population, early identification and treatment of bio-psychosocial risk 10 (range 8.9%-22.1%), early identification and treatment of urinary tract infections 4 (range 5.7%-41.6%), and adequate detection and treatment of gestational diabetes (range 0.9%-3.2%) (Table 3).

**Table 2.**
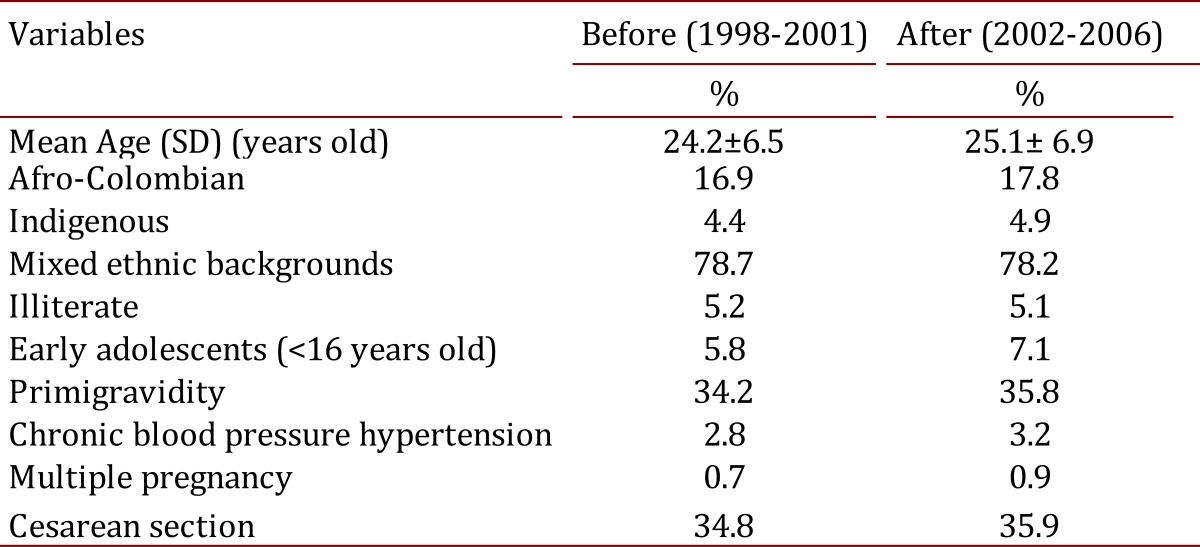
Socio-demographic and obstetric characteristic of Colombian pregnant women 1998-2006.

Pregnant women with pre-paid medical insurance who had access to early prenatal care had the lowest incidence of preeclampsia (range 0.4%-1.4%); pregnant women without medical insurance (low socioeconomic status) had a greater incidence of preeclampsia (range 1.4%-3.2%). The application of BPSM was influenced mainly by superimposed preeclampsia versus preeclampsia de *novo*. 

**Table 3.**
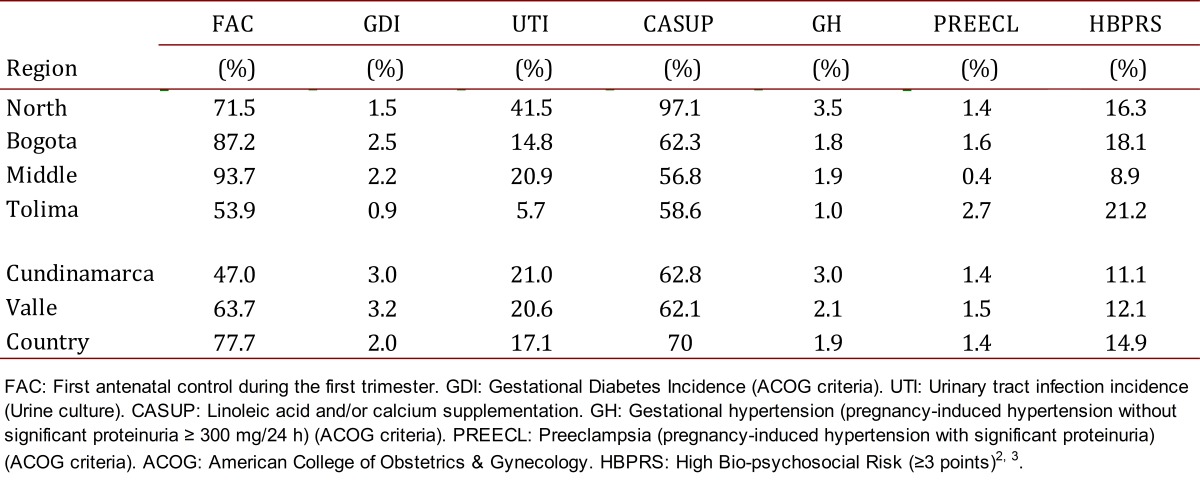
Biomedical interventions to reduce preeclampsia risk by Colombian regions 2002- 2006.

Macrosomia (birth weight > 90^th^ percentile for gestational age) has had a significant reduction in the incidence (5.1% to 4.2% ,OR = 0.816, 95%CI= 0.809 - 0.822) (Table 4). 

**Table 4.**
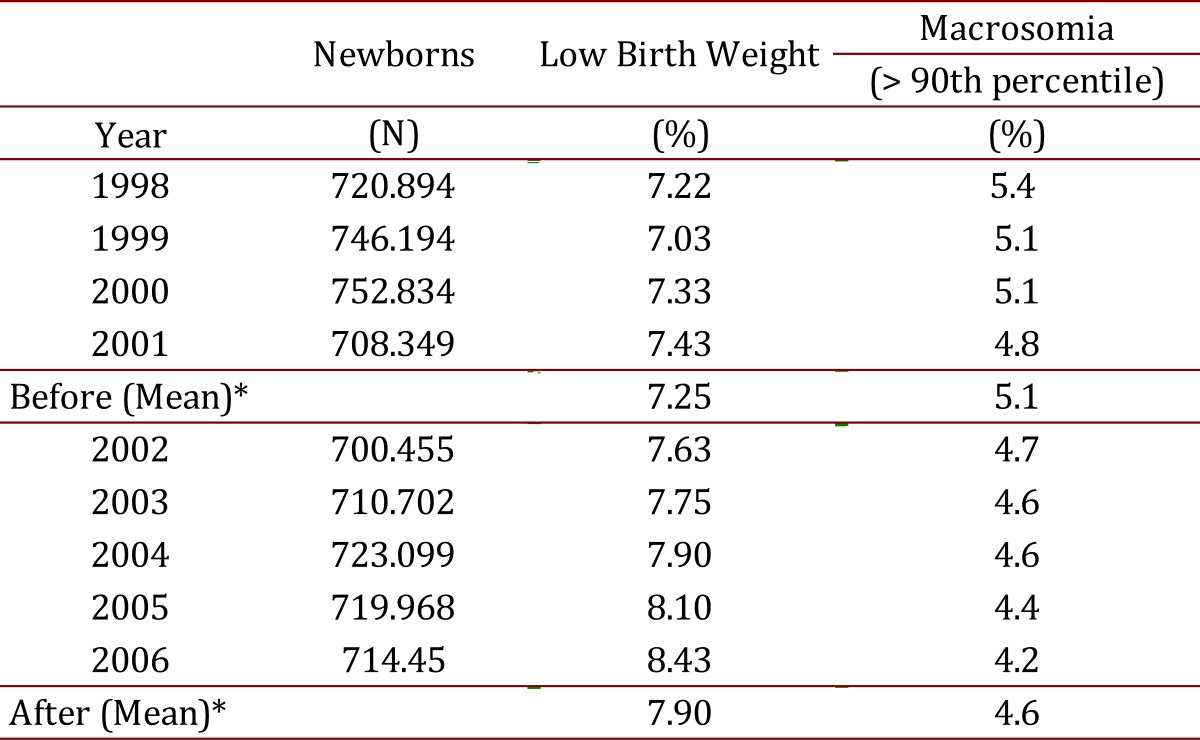
Birth weight with the introduction of a biopsychosocial model (2002-2006).

Six years after the implementation of BPSM (2002-2006) the general maternal mortality was reduced 23% (OR=0.77, CI 95% 0.71-0.82). The slight reduction in birth rates during the period was not associated with the reduction in general maternal mortality (*p*= 0.83). 

**Figure 1.**
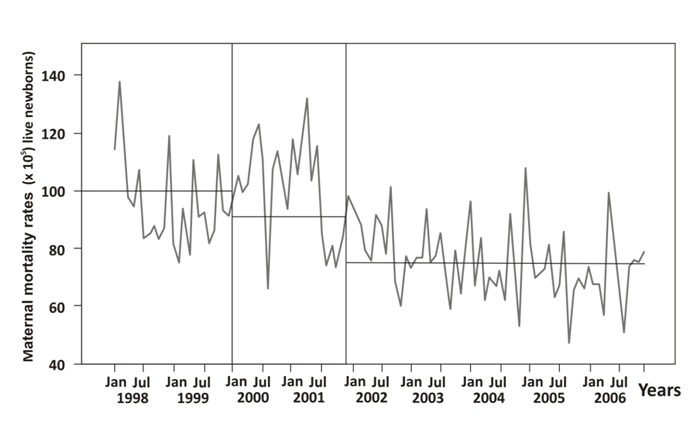
General maternal mortality. Colombia 1998-2006

With the introduction of obstetric protocols (2000-2001), changes in the ratio of maternal mortality was insignificant, in addition with the introduction of BPSM (2002-2006) the reduction in general maternal mortality decreased to 23% (Fig. 1) (interrupted time series analysis, Chi-square= 83.61, *p*= 0.024) (Table 5), same results were observed with the specific maternal mortality by preeclampsia (3.6% to 45.0% (median: 28.9 x 10^5^ versus 15.9 x 10^5^ live newborns) (interrupted time series analysis, C= 41.40, *p* = 0.968) (Table 5).

**Table 5.**
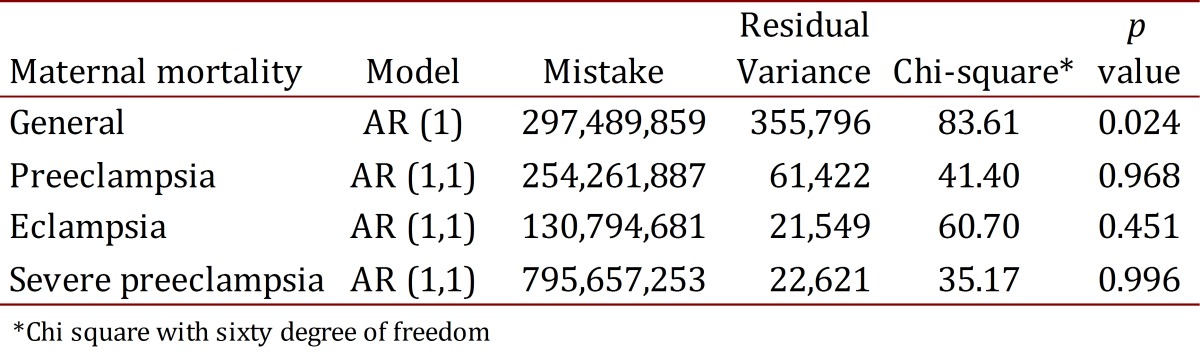
Interrupted time series analysis. Colombia 1998-2006

The reduction of complications of labor was of 34.4% (median: 8.6 x 10^5^ versus 6.4 x 10^5^ live newborns), by hemorrhage (postpartum, placenta preview and abruption of placenta) of 25% (median:11.3 x 10^5^ to 8.5 x 10^5^ live newborns), by embolism of 3.7% (median 2.8x 10^5^ versus 2.7x 10^5^ live newborns), by sepsis of 2.0% (median 10.2x 10^5^ versus 10.0x 10^5^ live newborns), and by thrombophilia of 1.7% (median 2.87x 10^5^ versus 2.82x 10^5^ live newborns).

Incidences of maternal mortality by preeclampsia had a significant reduction (median: 28.9 versus 15.9 x10^5^ live newborns) and by macrosomia (birth weight > 90^th^ percentile for gestational age had a significant reduction ( 5.1% versus 4.2%, respectively) (Table 4). The maternal mortality rate due to labor complications as cephalopelvic disproportion, cesarean section and hemorrhage was correlated with the incidence of preeclampsia (adjusted R^2^= 0.62, CI95%: 0.27-0.94, *p*= 0.006). 

Multiple linear regressions showed that maternal mortality by preeclampsia was not associated to early prenatal care (first trimester), early identification and treatment of urinary tract infections, nor to early treatment of gestational diabetes (second trimester) (*p*= 0.11). Furthermore, the above interventions together with calcium supplementation were not correlated to maternal mortality by preeclampsia (*p*= 0.24). However, a significant correlation was observed with the above interventions together with nutritional supplementation 2 ^-^ 7 and improved social support (BPSM) (Table 2) (*p*= 0.042).

## Discussion

**Figure 2.**
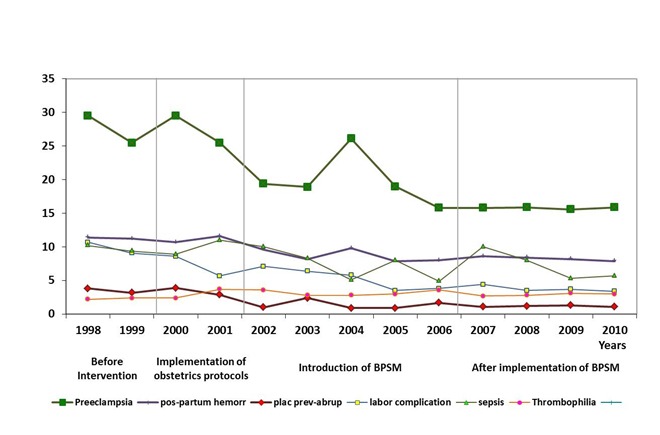
Maternal mortality rates by causes. Colombia 1998-2010

The main cause of maternal mortality in developing countries is preeclampsia, which increases perinatal mortality by fivefold. The problem is of greater concern when we consider that maternal mortality in Colombia is four times higher than in developed countries 1. After five years of implementation of BPSM the general maternal mortality remained reduced by 23% (*p*= 0.024) (Fig. 1), interestingly by preeclampsia (Fig. 2).

This study was conducted during a nine-year period (four years before and five years after BPSM) using a dual notification system for epidemiological surveillance of maternal deaths and with a similar system to guarantee the quality of medical care. Prior to 1998, the country only had one epidemiological maternal-death surveillance system. This caused an important bias resulting in under-reporting of maternal deaths that is not found in today's dual-notification system. Bias was controlled with this dual surveillance system.

A potential confounder was the impact of the small reduction in the number of newborns due to family planning on maternal mortality during this time period; this reduction was not correlated to maternal mortality. Another potential confounder was poor quality in medical care which was well-observed and controlled. The highest proportion of biomedical interventions occurred early in prenatal care (Table 4).

The observed results were attributed to the implementation of obstetric protocols with nutritional and psychosocial interventions (BPSM). Calcium supplementation alone does not reduce free intracellular calcium and does not improve endothelial function, which can be improved with the combined effect of other nutritional factors^6^^,^ 7 ^,^ 10. Urinary and periodontal infections and gestational diabetes are related to preeclampsia risk associated with oxidative stress and endothelial dysfunction 11. Early detection and early treatment can decrease endothelial injury 4. Enhancing endothelial function reduces multisystem failure, which is the most common cause of maternal death associated with preeclampsia. 

Currently, preeclampsia prevention is not possible. This is especially true in developing countries where half of the population is poor and where there are nutritional and psychosocial disadvantages. The Colombian social security system guarantees access and quality in prenatal and hospital care. It is widely accepted that the best hospital care reduces maternal mortality due to preeclampsia, complications of labor, hemorrhage, sepsis, and thrombophilia 1. As was observed in this study, general maternal mortality was reduced by 9.0% (Fig. 1) and specific maternal mortality by preeclampsia was reduced by 3.6% (Fig. 2) after the introduction and implementation of the obstetric protocols.

An increased number of health teams in the country were trained and monitored. The BPSM has biological plausibility for reducing preeclampsia risk 2 ^-^ 8 ^,^ 12, as was demonstrated in different studies and with different national and international populations. Endothelial dysfunction is an early condition present in pregnant women who later develop preeclampsia. It can develop from nutritional deficiencies, metabolic disorders, psychosocial stress, or asymptomatic infections. Inflammation and oxidative stress could be underlying mechanisms that greatly contribute to the development of insulin resistance associated with preeclampsia 13. The BPSM included early identification and modification of factors with potential influence on endothelial function 14 ^,^ 15 (Table 2).

Healthy pregnant women present a certain degree of inflammation and preeclamptic women have an excessive inflammatory response 16. Interestingly, high stress and low social support during pregnancy decreases cellular immunological response 17, increases C-reactive protein IL1β and IL6 18 ^-^ 21, and reproduces PE in animals 22 (exclusively human disease). The role of these cytokines during normal pregnancy is not entirely understood. Some have been related to the mechanisms involved in the initiation and maintenance of gestation. For instance, tumor necrosis factor (TNFα) and natural killer cells appear to regulate the invasion and growth of trophoblast in maternal and spiral arteries 14. The stimulation of the sympathetic nervous system can alter NK cell function, facilitating poor invasion of extra-villous trophoblast 23. The role of psychosocial factors in preeclampsia is a growing development 22 ^,^ 24 ^-^ 26. The impact of psychosocial factors on the pathogenesis of cardiovascular disease is well documented 25 ^,^ 27.

Blood pressure assessment, proteinuria screening, and early termination of pregnancy have been successful means to reduce maternal mortality by preeclampsia as was observed with the introduction of the obstetrics protocols (3.6%) (Fig. 1). We believe that the high prevalence of diseases or conditions associated with oxidative stress and endothelial dysfunction can increase the probability of having a multisystem failure if preeclampsia is present. In developing countries the most common cause of maternal mortality by preeclampsia is from a multisystem failure.

A decrease in maternal mortality could be related to the action of many factors and many were intervened with the implementation of obstetric protocols (2000-2001) (Figs. 1 and 2); however, with the introduction of BPSM (2002-2006) the reduction in general maternal mortality increased by three times (Fig. 1); interestingly after the implementation of BPSM the reduction of maternal mortality by preeclampsia increased by seven times. The implementation of the National Public Health Plan (Decree 3037 Ministry of Health and Social Protection) (2007-20210) included the BPSM and the maternal mortality by preeclampsia remained low (Fig. 2).

Today, prevention of preeclampsia is not possible; however, a reduction in maternal mortality by preeclampsia was observed with the introduction and implementation of obstetric protocols and BPSM. The reduction in maternal mortality by postpartum hemorrhage was associated with the implementation of red code. The national experience of a reduction in maternal mortality due to preeclampsia in a developing country is the main contribution of this study. There are clear implications for regional public health care. 

A limitation of the study is the fact that observational prospective studies can be biased; however, randomized double-blind controlled trials are not possible due to ethical considerations. Community work to evaluate the impact of this model in other populations is still being done.
